# In what ways does placeness affect people’s behavior? Focusing on personal place attachment and public place image as connecting parameter

**DOI:** 10.3389/fpsyg.2024.1394930

**Published:** 2024-06-18

**Authors:** Phengsy Dalavong, Ha Na Im, Chang Gyu Choi

**Affiliations:** ^1^Department of Urban and Regional Development Management, Hanyang University, Seoul, Republic of Korea; ^2^Big Data Lab, RealtyPlanet Co., Ltd., Seoul, Republic of Korea

**Keywords:** placeness, behavioral intention, place attachment, place image, path analysis

## Abstract

Placeness is believed to play a significant role in enhancing the well-being and place-use of individuals, contributing profoundly to how spaces are experienced and interacted with. Despite its perceived importance, there is ongoing debate and insufficient clarity about how exactly placeness influences people’s behavior. This study aims to bridge this gap by theorizing and investigating the pathways from placeness to people’s behavioral intentions, emphasizing the roles of personal place attachment and public place image as pivotal mediators in this relationship. To explore these dynamics, we conducted a survey in Japan, examining the complex interplay between placeness and behavioral intentions, given their rich cultural heritage and modern urban pressures. We employed the Partial Least Squares Structural Equation Modeling (PLS-SEM) approach for path analysis. The analysis suggests that placeness influences behavioral intention through personal place attachment. While placeness does affect public place image, this public image does not have an impact on behavioral intention. The results demonstrated that an individual’s activities, experiences, and cognition of a place are significant factors in creating the intention to engage in word-of-mouth, recommendation, and revisiting behaviors. Policymakers, urban planners, and designers need to understand how to foster people’s behavioral intentions when creating a place imbued with placeness.

## 1 Introduction

The urban built environment or physical aspects of a place have been confirmed by numerous studies to significantly impact human behavior (Jacobs, 1961; Halpenny, 2010; Relph, 2015). Consequently, many urban planners and designers are increasingly focusing their attention and investments on the development of these places. However, the mechanisms through which these places influence behavior remain a topic of debate. Indeed, it is widely accepted that the physical environment alone is insufficient to account for these effects, as they are also influenced by human perception, a concept that is well-established in environmental psychology. This study aims to theorize and provide empirical evidence on the structure and pathway from placeness to people’s behavioral intentions, by focusing on personal place attachment and public place image as mediators.

Since the 1970s, the study of placeness has attracted considerable interest, distinguishing between space and place (Relph, 1976; Tuan, 1977). Research has explored the relationship between humans and their environments from various perspectives, including place and migration (Rishbeth and Powell, 2013; Westin, 2016), place and tourism (Pike, 2002; Ge et al., 2022), environmental and architectural psychology (Donald, 2022), and place in environmental psychology (Lewicka, 2011). These studies have provided empirical evidence on how place influences behavior, such as showing that place image significantly affects residents’ attitudes toward tourism (Stylidis, 2016), and place attachment influences pro-environmental behavior (Halpenny, 2010; Ramkissoon et al., 2012; Dang and Weiss, 2021). Additionally, small sports events have been found to impress visitors, enhancing their intention to revisit and recommend a location (Su and Hsu, 2019).

Research in urban studies and architecture has also attempted to understand how the environment influences behavior. This includes methods such as sketching and mapping people’s behaviors (Lynch, 1964; Whyte, 1980), exploring how individuals cognitively differentiate places (Schertz et al., 2022), and studying multisensory perceptions in various (Lucas and Romice, 2010). These methods and results are effective in capturing an individual’s current perceptions and interactions with places, highlighting how personal perceptions of places influence their behavior and intentions. Meanwhile, behavioral studies underscore the significance of public perception and social norms in shaping behavior, as illustrated by Ajzen (1991). Tourism research further emphasizes the importance of the image of a place in influencing visitors’ willingness to visit places, highlighting public perceptions of places. Therefore, these studies highlight the important roles of personal and public perceptions in connecting placeness to behavioral intention.

Despite these advancements, previous research has been limited to two perspectives. Firstly, studies often examine the influence of place on behavioral intention in isolation, primarily focusing on either personal place attachment (Vaske and Kobrin, 2001; Halpenny, 2010) or public place image (Elliot et al., 2011; Stylidis et al., 2016). Secondly, while many studies focus on contemporary events, few explore the effects of placeness on the behavioral intentions of visitors in urban communal life contexts. To address these gaps, this study investigates the pathways through which placeness influences people’s behavioral intentions. We have developed a theoretical framework and structured hypotheses to analyze both the direct and indirect relationships between placeness and behavioral intention, considering factors such as place attachment and place image. Employing Partial Least Squares Structural Equation Modeling (PLS-SEM), we have chosen commercial districts in Tokyo, Kyoto, and Osaka as our survey sites. These locations offer urban environments that reflect a blend of tradition and modernity, making them ideal contexts to explore how historical and contemporary placeness influences visitor behaviors in communal life.

## 2 Literature review

### 2.1 Placeness and behavioral intention

The term “placeness” carries a complex meaning and frequently overlaps with other place-related concepts. In this paper, we narrow down the definition of “placeness.” We define it simply as a place that is not only in terms of its physical uniqueness or characteristics but also embodies the relationship between humans and place, considering the placeness as a whole. This definition aligns with Relph’s (2015) conceptualization of placeness as a “conveniently broad term that allows [one] to consider everything to do with the diverse qualities, interpretations, uses, and experiences of place, from place cells in the hippocampus to a global sense of place.”

The impact of place on behavioral intention has been widely studied in urban studies. On one hand, towns or places created solely with a focus on economic factors—such as rapid development, modernization of buildings, and mass infrastructure—may result in cities lacking variety (Jacobs, 1961). This can lead residents to experience a sense of unlivability and placelessness. For instance, Relph’s (1976) work on “Place and Placelessness” highlights how post-industrial urban development diminishes the sense of placeness in cities. He argues that mass production and standardization in urban planning have led to a homogenization of cities, fostering a sense of placelessness (Relph, 1976). This argument is also supported in urban history. Consequently, urbanists and architects have proposed a new planning and design agenda focused on people. They believe that the design of public spaces can significantly impact individual behavior, affecting social interaction (Jacobs, 1961; Whyte, 1980; Gehl, 2013). One influential work in this area is Whyte’s (1980), which identifies several key characteristics of successful public spaces, such as ample seating, natural sunlight, and opportunities for social interaction. In his work “Cities for People,” Gehl (2013) emphasized that cities should be designed to promote social interaction, pedestrian activity, and public space, focusing on the importance of creating physical environments that suit the human scale for optional and social activities. This highlights how physical environments are crucial in shaping human behavior.

On the other hand, despite the mass of buildings and poor environmental conditions, residents who live in these areas still feel attached to their place and revisit them. Why does such behavior persist? This question motivates our study. Many studies have attempted to describe this phenomenon, suggesting that attachment may be due to personal experiences, memories, and social interactions within the place (Livingston et al., 2010; Scannell and Gifford, 2017), indicating a tight correlation to the places, or past experiences which serve as mediators between the physical environment and behavior. In other words, the influence is not only direct but also indirect. Several empirical studies in heritage and cultural sites have confirmed that place influences the behavioral intention of residents and visitors through both direct and indirect effects (Im et al., 2013; Zhang and Li, 2021; Zhang et al., 2021). In environmental psychology and built environment behavior studies, a more complex mechanism of how place influences behavioral intention is proposed. For example, Ajzen (1991) defines behavioral intention as an individual’s readiness to perform a given behavior, influenced by their attitude toward the behavior, subjective norm Ajzens, and perceived behavioral control.

In this sense, physical environment attributes may not fully explain the influences on behavior, suggesting that a more comprehensive structure is needed. Therefore, to understand the effect of placeness on behavior, it’s necessary to look beyond the direct effects and consider a comprehensive structure that includes both direct and indirect effects. Based on the literature, we consider two factors as mediators between placeness and behavioral intention: personal perception factors in the form of ‘place attachment’, which focuses on individual preferences, past experiences, and attitudes; and public perception factors, embodied in the ‘public image’ of the physical environment and social norms. The subsequent section will discuss personal place attachment and public place image in more detail.

### 2.2 Place attachment and place image

The relationship between individuals and their environments, particularly “Place Attachment,” has been widely studied in environmental psychology (Altman and Low, 1992; Hidalgo and Hernández, 2001; Scannell and Gifford, 2010; Lewicka, 2011). Many researchers have delved into the process of an individual’s cognitive relationship with a place through theoretical frameworks. For instance, the tripartite organizing framework of Scannell and Gifford (2010) illustrates the relationship between the person, processes, and place. Additionally, Gustafson (2001) suggests a framework that views a place’s meaning through the lens of self, environment, and other aspects. Therefore, place attachment can be viewed as an individual’s perception formed by the interaction of a person’s feelings, the function of place, cognition, experience, and beliefs after experiencing the essence of a place.

Studies of place attachment have shifted toward more empirical work and are widely conducted in many fields. Particularly, the testing relationship between place attachment and behaviors such as willingness to pay, loyalty, risk-coping behavior, tourism management, civic engagement, pro-environmental behaviors, and pro-tourism behavior (Vaske and Kobrin, 2001; George and George, 2004; Gross and Brown, 2008; Halpenny, 2010; Lewicka, 2011; Ramkissoon et al., 2012, 2013; Silva et al., 2013; Su and Hsu, 2019; Dang and Weiss, 2021; Wan et al., 2022). Some of these studies confirmed that place attachment has a positive and direct effect on behavior, forming a bond between an individual and a specific place (e.g., memories, emotions, identity). For example, a study on pro-environmental behavior highlighted place attachment as a personal factor positively influencing pro-environmental behavior, (Halpenny, 2010; Ramkissoon et al., 2013). Halpenny (2010) researched visitors to Point Pelee National Park in Canada and discovered a strong correlation between the intensity of place attachment and the prediction of place-related pro-environmental intentions. The study also highlighted the predictive power of place attachment to pro-environmental behavioral intentions in everyday life. Additionally, people who have developed a strong attachment to a place are more likely to protect the environment of a park in Australia (Ramkissoon et al., 2012). In other fields such as tourism management, studies have found that place attachment positively influences behavior intention in terms of word of mouth, revisiting, and active involvement in activities (Su and Hsu, 2019; Soonsan and Sukahbot, 2020). Research in the field of urban studies has found similar results. For instance, in urban areas in South Korea, it was shown that people who have developed an attachment to a place tend to have positive word of mouth, recommend the place to others, and revisit the place in the future (Kim and Choi, 2011; Seong et al., 2016; Son et al., 2023).

Not only does personal place attachment influence behavioral intention, but public place image perception is also a crucial factor. Place image, which focuses on the image of a place, particularly regarding its common and well-known characteristics, also has a relationship with behavioral intention. Place image is shaped by social factors and the meaning attributed to the place by the community (Kang and Choi, 2012; Im et al., 2013). Additionally, Relph (1976) emphasizes that the meaning people give to a place is important in understanding their perception and experience of it. This relationship between place image and behavioral intention has been studied extensively in the tourism sectors using the terminology “Destination Image.” Previous studies have shown mixed results regarding the relationship between place image and behavior. The research on overall destination image, event, and festival image has demonstrated that it influences behavioral intentions to choose a destination, with both direct and indirect effects (Chen and Tsai, 2007; Chi and Hailin, 2008). Papadimitriou et al. (2015) found that cognitive and affective place image components positively influenced residents’ word-of-mouth intentions toward tourists in the urban area of Athens, Greece. Moreover, a study by Chi and Hailin (2008) found evidence of an indirect relationship between destination image and destination loyalty in the context of the state of Arkansas—Eureka Springs. In aspects of urban planning and design, the public perception of a place is not only organically bound by itself. It is also affected by urban planner-designer work that attempts to create an image of the place that refers to a mental map of the city, to make the place easy to memorize as seen in detail in The Image of City (Lynch, 1964).

The studies mentioned above recognize concepts such as place attachment, place image, and behavioral intention for their intrinsic value and contribution to city development. However, to genuinely unlock the potential of urban development and foster more comprehensive improvements in our cities, it is imperative to integrate these concepts.

## 3 Hypothesis and methodology

### 3.1 Hypothesis setting resource

This study aims to address the gaps in prior research by proposing a comprehensive structural pathway. We seek to integrate concepts such as placeness, place attachment, and place image to understand their influence on behavioral intention. Notably, we incorporate place attachment and place image as mediators, bridging the connection between placeness and behavioral intention. To delineate this comprehensive pathway from placeness to behavioral intentions, we have pinpointed five direct pathways. Among them, three pathways directly map the transition from placeness, place attachment and place image, to behavioral intention. The remaining two pathways explicitly connect placeness with place attachment and place image, respectively. Furthermore, we have identified two indirect pathways linking placeness to behavioral intention via place attachment and place image.

Placeness is the initiative of the awareness of the people who perceive the place. and it considers in many inspects those visitors and residents perceived environment surrounding in a single element. Therefore, the relationship started from the perceived placeness that has directly affected the personal place attachment, public place image, and behavioral intention. Therefore, we assumed the hypotheses below:

*H1*: Placeness (PL) has a positive direct effect and is significant for place attachment (PA).

*H2*: Placeness (PL) has a positive direct effect and is significant for place image (PIM).

*H3*: Placeness (PL) has a positive direct effect and is significant for behavioral intention (BI).

Additionally, place attachment and place image itself we also assumed that it is have positive and direct effects on behavioral intention. as studies on place attachment in psychology studies being used to represent the connection between place and behavior through psychological processes. This process is often linked to individual cognition and past experiences and can influence future behaviors (Halpenny, 2010; Ramkissoon et al., 2013; Dang and Weiss, 2021). In tourism studies the public image is used to describe people’s perceptions of a place collectively and much research also shows the connection between the place image and the future behavior of both residents and visitors. For more details see (Chi and Hailin, 2008; Elliot et al., 2011; Papadimitriou et al., 2015; Stylidis et al., 2016). Therefore, we assumed that two place attachments and place image directly influenced the behavioral intention as the hypotheses below:

*H4*: Place attachment (PA) has a positive direct effect and is significant for behavioral intention (BI).

*H5*: Place image (PIM) has a positive direct effect and is significant for behavioral intention (BI).

**Figure 1:**
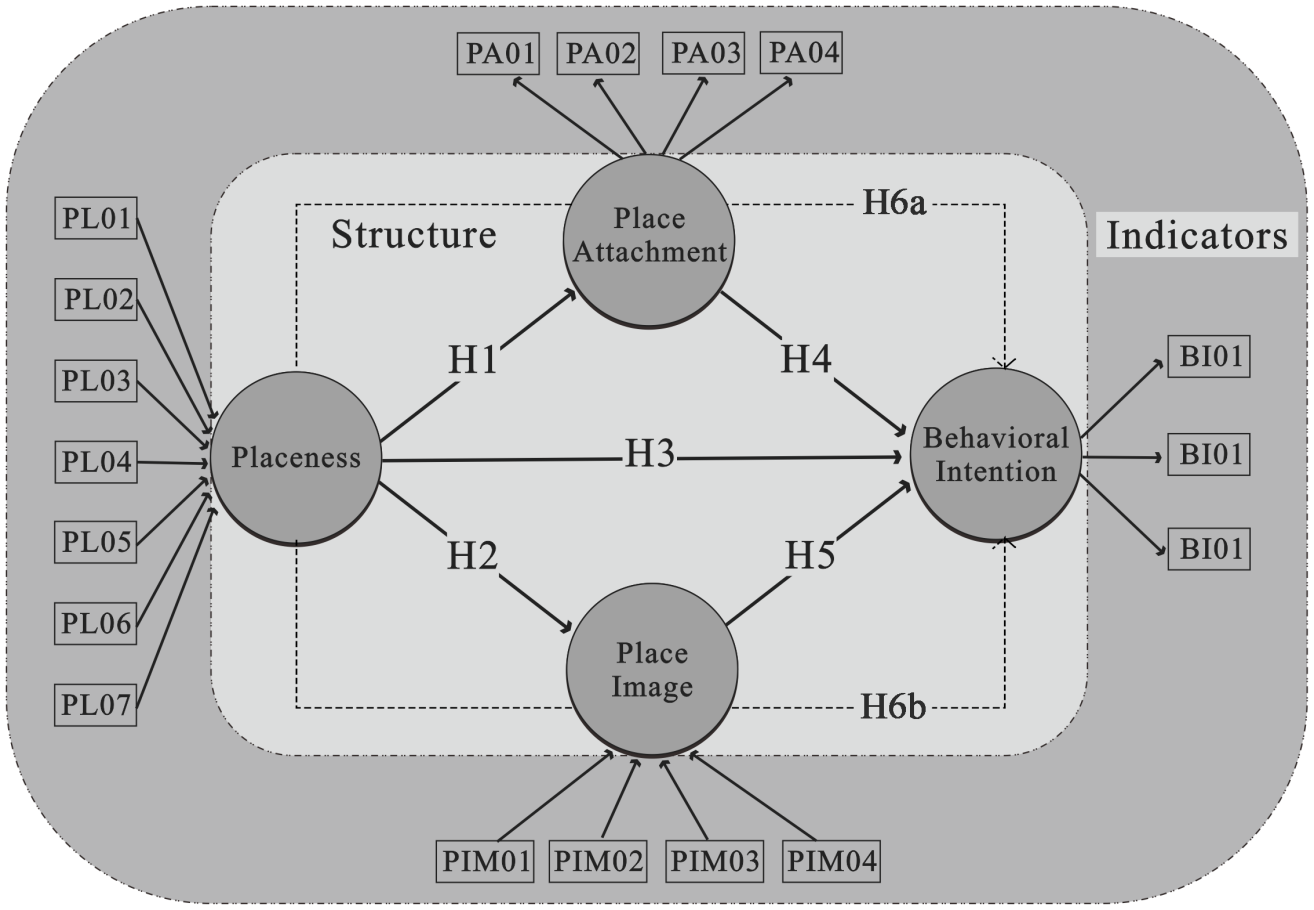
Theoretical framework.

Nonetheless, this study employs an empirical approach and utilizes hypothesis testing to analyze the relationship between placeness and behavioral intentions. Based on previous literature reviews, it is hypothesized that placeness also has an indirect relationship with behavioral intentions through place attachment and place image. To investigate and confirm these relationships, this study proposes hypotheses 6a and 6b as the hypotheses below, and the overall structure framework is present in Figure 1.

*H6a*: Placeness (PL) has a positive indirect effect and is significant for behavioral intention (BI) through place attachment (PA).

*H6b*: Placeness (PL) has a positive indirect effect and is significant for behavioral intention (BI) through place image (PIM).

### 3.2 Measurement

A review of previous studies is required to develop the measurement of our proposed theorize framework. The measurement consisted of questions developed to capture how the respondents perceived the place and their behavior, see Table 1. For instance, the studies of placeness aim to uncover the definition and measurement of placeness from the perspective of urban residents and visitors. Which is not only focused on the uniqueness of the place, but it considers as a whole the characteristics of the areas. In the placeness construct see more details from Kang and Choi (2012) and Im et al. (2013) who widely studies measurement placeness. Additionally, place attachment has been widely studied in psychological environments that measure on the individual level through the place. For example, Williams and Roggenbuck (1989) and Kyle et al. (2005) have discussed and tested both place identity and place dependence to capture the function of place and personal perceptions of place. Place images are widely studied in tourism studies such as Echtner and Brent Ritchie (1993) and Kang and Choi (2012) and we focus on measurements of the social or public perception of places. Lastly, measuring behavioral intention is more common than studying universal behavioral intentions. Particularly, this approach is beneficial for scholars focusing on future behaviors related to places and tourism, as well as for organizers evaluating future behaviors after individuals have experienced certain events or visited certain places.

**Table 1:** Measurements of variables.

Construct and measure variables	
Placeness	
PL01	This place is funny, exciting, cheerful, creative, artistic, and dynamic
PL02	This place is crowded, complicated, and noisy
PL03	This place is various, free, new, and special
PL04	This place is intense, sophisticated, flashy, and meaningful
PL05	This place is unique, outstanding, and impressive
PL06	This place is beautiful, chaotic, and charming
PL07	This place is messy and promiscuous
Place image	
PIM01	This place for art and culture-oriented
PIM02	This place with various commercial facilities
PIM03	This place for taking a rest and walking
PIM04	This place working-oriented people
Place attachment	
PA01	I feel attached to this area
PA02	I will miss this area if it changes
PA03	I am attracted to the physical environment and atmosphere of this area
PA04	I feel more satisfaction here than in other places
Behavioral intention	
BI01	I will continue to revisit this area
BI02	I will tell others positively about this area
BI03	I will recommend it to those who want to visit this area

### 3.3 Data collection

Given the diverse nature of metropolitan regions, the selection of a specific area to represent an entire city can be quite varied. In this study, we selected distinct locales within three major Japanese cities – Tokyo, Kyoto, and Osaka – to validate the concept of placeness.

In Tokyo, our focus was on Shibuya, a vibrant retail and entertainment district renowned for its iconic “scramble” crossing and towering digital billboards. In Osaka, we concentrated on Dotonbori, a favorite tourist hotspot recognized for its bustling ambiance along the Dotonbori Canal in the Namba district. Meanwhile, in Kyoto, our attention was on Gion, a quintessential district celebrated for its rich historical culture, most notably the presence of Geisha and traditional Japanese eateries and boutiques lining Hanamikoji Street.

In conducting this study, we adhered to the general principles of ethical research, which are appropriate for studies classified as minimal risk. Our study involved distributing questionnaires and did not collect identifiable personal information from participants, thereby posing minimal or no risk to them. Consistent with ethical research practices, we ensured informed consent, maintained participant anonymity, and protected data privacy throughout the research process. Data collection took place from June to July 2013. Interviews were conducted between 6 p.m. and 10 p.m. Each questionnaire required approximately 5–8 min to complete. To accommodate our target audience, the questionnaire was translated into Japanese. It was divided into three sections. Part 1 emphasized respondents’ demographic and mobility traits, such as gender, age, occupation, and time spent on transportation. Part 2 touched upon the perceived reason for visiting, the facilities used, and the motivation behind their visit. Part 3 homed in on four primary factors: perceived placeness of the location (PL01-PL07), place attachment (PA01-PA04), place image (PIM01-PIM04), and behavioral intention (BI01-BI04). Both Parts 2 and 3 utilized a 5-point Likert scale, ranging from 1 (strongly disagree) to 5 (strongly agree).

### 3.4 Analysis method setting

Based on our literature review on the recognition of placeness, we identified both place attachment and place image as mediators. These serve as the connecting links between placeness and behavioral intention. This study employs PLS-SEM to explore the relationships among these four constructs. We chose PLS-SEM because it sheds light on the connections among multiple variables, examining the structure of interrelationships articulated through a series of equations, which are well-known for handling small sample sizes. Moreover, our decision to use the PLS-SEM approach was influenced by the fact that the constructs encompassed both formative and reflective measurements (Hair et al., 2020; Sarstedt et al., 2020). Within these constructs, placeness (PL) and place image (PIM) are categorized as formative, while place attachment (PA) and behavioral intention (BI) are classified as reflective.

Consequently, we adopted a two-step PLS-SEM modeling approach. First, in the measurement model, our focus was on the quality of indicators. For formative constructs, we examined the variance inflation factor (VIF), outer weight, and outer loading (Sarstedt et al., 2020; Hair et al., 2021). For reflective constructs, evaluation metrics such as Cronbach’s alpha, the average variance extracted (AVE), composite reliability (CR), and the heterotrait-monotrait ratio of correlations (HTMT) were utilized (Chin, 2010). Second, delved into the structural model, analyzing collegiality through the VIF, the R-squared value, the Q-squared value, and the path coefficient (Sarstedt et al., 2020).

## 4 Results

### 4.1 Descriptive analysis

Out of the 130 survey responses we received, 117 were fully validated, yielding an effective response rate of 90%. The characteristics of the respondents are detailed in Table 2. The respondents from these three areas were predominantly female, representing 58.12% of the total, with an average age of 26.88. The largest occupational groups were service sector workers and students, making up 30.77 and 25.6% of respondents, respectively. Notably, 68.38% of individuals spent less than an hour traveling to participate in the survey, which is consistent with the urban nature of the areas, located in the heart of urban environments and easily accessible via transportation.

**Table 2:** Demographic profile of respondents (*n* = 117).

Characteristics		Mean / N	S. D. / percentage
Ages		26.88	7.27
Gender	Male	49	41.88%
	Female	68	58.12%
Occupation	Student	30	25.64%
	Office worker	11	9.40%
	Service sector	36	30.77%
	Manual worker sector	2	1.71%
	Profession	13	11.11%
	Others	25	21.37%
Time spent on transportation	Less than 1 h	80	68.38%
	1–2 h	28	23.93%
	More than 3 h	9	7.69%
Purpose of visiting	Have an appointment	2.726	1.495
	Performances	2.094	1.402
	Eating and drinking	3.085	1.512
	Shopping	3.103	1.476
	Take a rest	2.402	1.427
	To commute to school	2.231	1.673
	No special purpose	2.154	1.436
Using facilities	Cultural facilities	2.026	1.380
	Coffee shop	3.077	1.403
	Restaurants	3.350	1.416
	Liquor store	2.675	1.485
	Clothing and accessory stores	2.906	1.456
	Entertainment facilities	2.615	1.496
	Educational facilities	1.692	1.263
Reason for visiting	Atmosphere	3.393	1.358
	Famous place	3.573	1.328
	Reasonable price	2.761	1.330
	Convenient for transportation	3.333	1.402
	Commercial facilities	3.718	1.286
	Near home and workplace	2.769	1.589

As for the nature and context of the commercial areas we surveyed, respondents reported visiting these areas due to the commercial facilities, which align with convenient transportation options and a pleasant atmosphere. The purpose of respondents visiting these areas is to utilize facilities that are well-provided, such as restaurants and coffee shops. They use these facilities primarily for eating, drinking, and shopping. These reasons for visiting were influenced by individual experiences. However, it is interesting to note that these areas are also visited because they are famous places, known for their social or public image.

These demographics and reasons for visiting these areas provide the fundamental context of the visit. Although other areas also offer these basic facilities, the reasons why visitors choose these areas and are willing to revisit, recommend them to others, and even return multiple times are the main interests of this study. The next section will show how the ‘placeness’ impacts behavioral intentions from an urban planning and design perspective, through the psychological perception of place.

### 4.2 The measurement assessment

Our theoretical framework incorporates two types of constructs: reflective and formative. To assess these constructs requires different methodologies (Hair et al., 2020, 2021; Sarstedt et al., 2020) detailed in Table 3. For the reflective constructs, such as place attachment and behavioral intention, we begin by evaluating the loading and significance (*p*-value) of each indicator. Place attachment is measured using four indicators: PA01, PA03, and PA04 each show an outer loading exceeding the 0.70 threshold, while PA02 (“I will miss this area if it changes”) has a loading of 0.645. Despite being below the typical cutoff, its significance at *p* < 0.05 justifies its retention in our model. Similarly, all indicators of behavioral intention surpass the threshold of 0.707, affirming their adequacy (Sarstedt et al., 2020).

**Table 3:** Measurement of talent variables and observed variables.

Construct	Measurement indicators				
Reflective construct	Indicators	AVE	CR	Cronbach’s alpha	Factor loadings
Place attachment		0.645	0.878	0.817	
	PA01				0.810***
	PA02				0.645***
	PA03				0.858***
	PA04				0.879***
Behavioral intention		0.659	0.852	0.743	
	BI01				0.721***
	BI02				0.837***
	BI03				0.870***
Discriminant validity (Heterotrait-Monotrait ratio)					
	Behavioral intention		Place attachment		
Behavioral intention	0.812				
Place attachment	0.571		0.803		
Formative construct	Indicators	VIF	Weights	T-Stat.	Factor loadings
Placeness	PL01	1.376	0.514**	5.560	0.717***
	PL02	1.309	−0.314*	2.111	−0.356*
	PL05	1.430	0.343*	4.708	0.640***
	PL06	1.404	0.369*	5.041	0.706***
	PL07	1.507	−0.114	2.066	−0.347*
Place image	PIM01	1.098	0.621**	5.142	0.799***
	PIM03	1.052	0.491**	3.945	0.661***
	PIM04	1.080	0.324	2.917	0.554**

***p < 0.001; **p < 0.01; *p < 0.05.

Next, we assess the reliability of these constructs using Cronbach’s alpha and Composite Reliability (CR), with both metrics requiring values above 0.70. Our findings indicate strong reliability for both constructs: place attachment (Cronbach’s alpha = 0.817, CR = 0.878) and behavioral intention (Cronbach’s alpha = 0.743, CR = 0.852), suggesting internal consistency within the measurement model (Hair et al., 2021). We then evaluate convergent validity through the Average Variance Extracted (AVE), with an acceptable threshold set at 0.5. The AVE values for place attachment (0.645) and behavioral intention (0.659) both exceed this benchmark, confirming adequate convergent validity. Discriminant validity is assessed using the Heterotrait-Monotrait ratio (HTMT). Our model meets these criteria as demonstrated in Table 3, with place attachment and behavioral intention showing an HTMT value under of 0.90, which supports their distinctiveness (Hair et al., 2017, 2021).

Turning to formative constructs, we first check for collinearity among indicators using the Variance Inflation Factor (VIF), with all indicators for placeness and place image recording VIF values below three, thus avoiding collinearity issues. The significance and relevance of the formative indicators were evaluated by analyzing their weight and loading using the bootstrap method with 10,000 replications. This method provided a robust and reliable evaluation of the indicators by resampling the data multiple times to account for the uncertainty in estimating weights and loadings (Sarstedt et al., 2020; Hair et al., 2021). The results showed that five formative indicators had insignificant weights and factor loadings below 0.5. However, two of these indicators were significant in terms of factor loading and were retained in the analysis (Sarstedt et al., 2020; Hair et al., 2021). Conversely, three formative indicators (PL02, PL03, and PIM02) were found to be insignificant (*p* > 0.05) and were removed from the model due to their factor loading values being far below the cutoff point. Therefore, after assessing all factors and constructs, the measurements are ready for evaluating the path and structural elements of our theoretical framework.

### 4.3 The structural model

We assessed the structural model by examining significant path coefficients, t-values, and explained variance (*R^2^* and *Q^2^*), employing a two-tailed bootstrapping method with 10,000 samples for hypothesis testing. This robust method addresses the uncertainties in model parameter estimation through repeated data resampling (Sarstedt et al., 2020; Hair et al., 2021). Unlike covariance-based SEM (CB-SEM), PLS-SEM does not utilize model fit indices like CFI or TLI, as it is variance-based and instead relies on *R^2^* and *Q^2^* values for assessing model fit (Hair et al., 2021).

Our analysis revealed no collinearity issues, with inner VIF values remaining below 3. The Standardized Root Mean Squared Residual (SRMR) was 0.090, indicating an acceptable fit (Hu and Bentler, 1999). The model’s goodness of fit was gaged by the strength of each structural path, showing notable effects on place attachment (*R^2^* = 0.246, *Q^2^* = 0.142) and place image (*R^2^* = 0.251, *Q^2^* = 0.161), as well as a collective impact of placeness, place attachment, and place image on behavioral intention (*R^2^* = 0.353, *Q^2^* = 0.126), as detailed in Table 4.

The primary goal of our study was to examine how placeness influences behavioral intention. The results of hypothesis testing showed that three out of five direct hypotheses had significant relationships. H1, asserting a positive effect of placeness on place attachment, was confirmed with a path coefficient (*β* = 0.469, *p* < 0.001). H2 demonstrated that placeness significantly affects place image (*β* = 0.501, *p* < 0.001). H4 established that place attachment directly enhances behavioral intention (*β* = 0.484, *p* < 0.001). Thus, H1, H2, and H4 were supported, highlighting significant pathways in these relationships, particularly from place attachment to behavioral intention.

Conversely, H3 and H5 were not supported. H3, which assessed whether placeness directly influences behavioral intention, showed a positive but insignificant effect (*β* = 0.203, *p* > 0.05), suggesting that placeness alone may not significantly affect future visitor behavior, aligning with findings by Kang and Choi (2012). H5, proposing a positive impact of place image on behavioral intention, showed a negative, insignificant effect (*β* = −0.033, *p* > 0.05), indicating that public perception alone might not significantly influence revisitation behavior, suggesting a reevaluation of the influence of initial visit decisions based on place image.

Finally, our study emphasized the mediating roles of place attachment and place image in the relationship between placeness and behavioral intention. The mediation analysis revealed a significant positive indirect effect via place attachment (H6a, *β* = 0.240, *p* < 0.001), supporting its role as a mediator. However, the indirect effect via place image (H6b) was negative and not significant (*β* = −0.016, *p* > 0.05), raising questions about the effectiveness of urban planning and design in shaping public perception and influencing revisitation behavior (Table 4).

**Table 4:** Hypothesis testing of structural model.

Hypothesis		β	T-Stat.	97.5% CI	Decision
Path coefficients direct effect					
H1	Placeness - > Place attachment	0.496 ***	6.649	[0.370, 0.641]	Supported
H2	Placeness - > Place image	0.501 ***	5.064	[0.370, 0.666]	Supported
H3	Placeness - > Behavioral intention	0.203	1.927	[0.017, 0.430]	Not supported
H4	Place attachment - > Behavioral intention	0.484 ***	4.867	[0.263, 0.653]	Supported
H5	Place image - > Behavioral intention	−0.033	0.330	[−0.221, 0.169]	Not supported
Path coefficients indirect effect					
H6a	Placeness - > Place attachment - > Behavioral intention	0.240***	4.024	[0.131, 0.361]	Supported
H6b	Placeness - > Place image - > Behavioral intention	−0.016	0.308	[−0.122, 0.090]	Not supported
Summary model		VIF	*R*	*R* ^2adj^	*Q* ^2^
Placeness		1.525			
Place attachment		1.422	0.246	0.239	0.142
Place image		1.433	0.251	0.245	0.161
Behavioral intention			0.353	0.336	0.126

***p < 0.001; **p < 0.01; *p < 0.05.

## 5 Discussions

The objective of our study was to delineate the influence of placeness on behavioral intentions. Our findings reveal that placeness does not exert a direct influence on behavioral intentions, thereby refuting H3. However, it does affect behavioral intentions indirectly through place attachment, which corroborates H6a. The analysis suggests that the concept of placeness, encompassing a diverse range of physical, social, and psychological aspects, is too multifaceted to directly impact visitor behavior. Instead, it significantly shapes individuals’ perceptions and emotional bonds with a location, indirectly influencing their behaviors. This aligns with prior research in the domain (Kang and Choi, 2012; Im et al., 2013).

**Figure 2:**
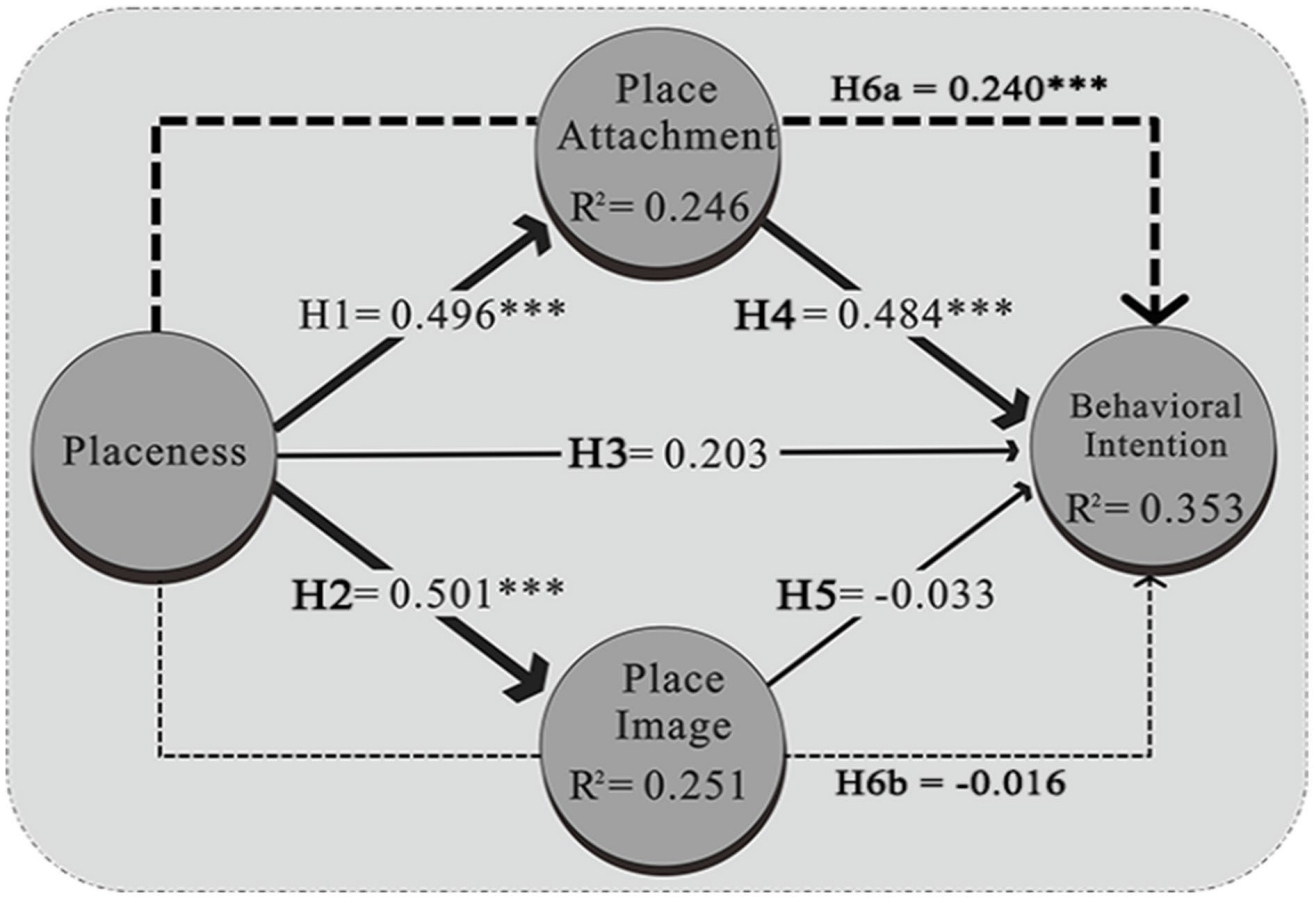
Results of PLS-SEM model.

Further examination into the roles of place attachment and place image revealed that both are critical in translating the effects of placeness into future behavioral outcomes at various levels. Specifically, place attachment directly influences behavioral intentions (supporting H4), echoing findings from numerous studies (Gross and Brown, 2008; Kang and Choi, 2012; Ramkissoon et al., 2013). Moreover, place attachment serves as a mediator between placeness and behavioral intentions, suggesting that strong attachments to a place enhance the likelihood of positive behavioral intentions, such as recommendations and revisits. This underscores the necessity for designing spaces and experiences that foster positive perceptions and attachments, thereby influencing visitor behaviors, see Figure 2.

Although placeness significantly influences the image of a place (supporting H2), its public image does not have a direct impact on behavioral intentions (rejecting H5). This finding aligns with previous research that underscores the importance of place image in fostering tourism (Shen et al., 2019). The public image of a place is shaped by a myriad of factors, including its physical and functional characteristics, as well as the meanings attributed to it by the community. Understanding the social context within which a place exists is vital for effectively shaping its public image. This understanding is crucial for tourism management strategies aimed at attracting tourists by promoting a positive image of a city as an appealing tourist destination. However, to foster behavioral intentions such as word-of-mouth promotion, making recommendations, and encouraging revisits, it is essential to engage with the individual feelings and experiences associated with placeness. This approach emphasizes the need to consider not just the broad characteristics that contribute to the place’s image, but also the personal connections and perceptions that visitors and residents form with the place.

Our focus on urban studies highlights the importance of balancing a city’s crafted image with the personal experiences of its residents, particularly through the lens of path coefficients in our analysis. Placeness predominantly influences place image (H2) over place attachment (H1) and does not impact behavioral intention through place image (H6b). This may indicate that urban planners and designers prioritize enhancing a place’s image to make it more appealing and unique, often overlooking the personal experiences of residents and visitors. These findings are invaluable for policymakers, planners, and designers, offering insights into how perceptions of placeness affect future behavioral intentions.

The implications for urban planners and government bodies in urban planning are significant, emphasizing the creation of livable and diverse cities that facilitate positive interactions among residents and their surroundings. Understanding the dynamics between placeness and behavior is pivotal in urban planning and development. By valuing residents and visitors’ personal perceptions, urban planners can design spaces that nurture positive interactions and emotional connections, contributing to the development of more sustainable and livable cities. This involves considering accessibility, functionality, esthetic appeal, and promoting community engagement and social interaction. It’s also critical to acknowledge that personal perceptions may be influenced by past experiences and beliefs, which should be integrated into planning and design strategies.

## 6 Conclusion

The aim of this study was to explore the influence of placeness on behavioral intentions within an urban context, focusing on the cities of Tokyo, Kyoto, and Osaka in Japan. Our model assessed both direct and indirect effects, with a particular emphasis on place attachment and place image as mediators between placeness and behavioral intentions.

The findings reveal that placeness does not directly influence behavioral intention; however, it exerts an indirect influence through place attachment. This indicates the importance of personal perceptions in fostering word-of-mouth promotion, recommendations to others, and a willingness to return to a place in the future. Among the factors examined—placeness, place attachment, and place image—only place attachment directly impacted behavioral intention. This outcome is pivotal for urban planning and design, underscoring that while place image contributes to a collective or public perception, it does not significantly influence future visitor behaviors.

Our analysis also showed that although placeness significantly affects place image, this does not translate into behavioral changes. This suggests that urban planners and policymakers might focus excessively on enhancing a location’s image without sufficiently considering individual experiences. Therefore, future urban development should prioritize diverse consumer groups and incorporate facilities that enhance personal experiences and emotional connections to the place. This approach should aim at creating memorable experiences and activities that encourage engagement and repeat visits.

Moreover, the study highlights the importance of tailoring urban development to the specific needs and behaviors of visitors, rather than merely improving physical aspects of the environment. Considering personal experiences during the planning and design process can lead to more effective urban spaces that meet the expectations and desires of users.

This study’s limitations are primarily associated with its narrow focus on urban settings during nighttime, underscoring the need for broader research that spans various times and contexts to fully understand usage behavior. Additionally, the limitation of the sample size may restrict the generalizability of the findings. Moreover, this study considered only a limited set of factors influencing the relationship between place attachment and place image. Future research should expand this scope to include variables such as cultural backgrounds and past experiences, which could provide deeper insights into the dynamics of place attachment and its impact on behavioral intentions.

## Data availability statement

The data presented in this study are available on request from the corresponding author.

## Ethics statement

Ethical review and approval was not required for the study on human participants in accordance with the local legislation and institutional requirements. Participants provided informed consent.

## Author contributions

PD: Formal analysis, Software, Visualization, Writing – original draft, Writing – review & editing. HI: Conceptualization, Methodology, Resources, Writing – review & editing, Data curation. CC: Conceptualization, Funding acquisition, Investigation, Methodology, Resources, Supervision, Writing – original draft, Writing – review & editing.
